# Engineered Trivalent Human IgG1-Fc Proteins for Potent Complement Inhibition

**DOI:** 10.3390/cells15131156

**Published:** 2026-06-25

**Authors:** Ian K. Campbell, Daniel Ortiz, Carlos Bosques, Matthew P. Hardy, Andrea Tester, Vesna Tomasetig, Daniel Couto, Thomas Gentinetta, Sabine Pestel, Padmapriya Ponnuswamy, Fabian Käsermann, Rolf Spirig

**Affiliations:** 1 CSL Limited, Bio21 Institute, 30 Flemington Rd., Parkville, VIC 3010, Australia; 2 Momenta Pharmaceuticals Inc., Cambridge, MA 02142, USA; 3 CSL Behring Biologics Research Center, Swiss Institute for Translational and Entrepreneurial Medicine, 3010 Bern, Switzerland; 4 CSL Behring GmbH, 35041 Marburg, Germany

**Keywords:** innate immunity, Fc receptors, complement

## Abstract

High-dose intravenous immunoglobulin (IVIG) is used to treat autoimmune and inflammatory diseases, and several studies demonstrate that the therapeutic effects of IVIG can be recapitulated with the fragment crystallizable (Fc) portion. Further, recent data indicate that recombinant multimeric Fc molecules exhibit potent anti-inflammatory properties. In this study, we investigated the biochemical and biological properties of different recombinant human IgG1 Fc molecules with increasing valency and avidity, combined with mutations to increase binding affinity to complement protein C1q. These molecules were investigated for their potential dual antagonism: to antagonize Fcγ receptor (FcγR) effector functions (Ab-dependent cellular phagocytosis) in vitro, and to inhibit the activation of the classical complement pathway. C1q-binding mutants demonstrated an exponential increase in potency to inhibit the classical pathway in correlation with increasing multimerization. Importantly, in contrast to other multimeric Fc constructs such as Fc hexamers, no generation of complement C4a was observed. Reducing the binding affinity to FcγRIIB resulted in a half-life extension of the trivalent hIgG1-Fc molecules in human neonatal Fc receptor transgenic (hFcRn Tg) mice. Our data demonstrate a potent anti-inflammatory effect of recombinant human IgG1-Fc C1q-binding mutants in vitro and in vivo, mediated by blockade of FcγRs and inhibition of complement activation.

## 1. Introduction

Plasma-derived IgG is used to treat primary and secondary immunodeficiency. For this purpose, IgG can be administered either intravenously as IVIG or subcutaneously as SCIG. IVIG/SCIG products are manufactured from pooled plasma of thousands of healthy donors, which ensures a diverse antibody (Ab) repertoire. IVIG has been increasingly used for the treatment of patients with chronic or acute autoimmune and inflammatory diseases, such as immune thrombocytopenia (ITP), Guillain–Barré syndrome, Kawasaki disease, chronic inflammatory demyelinating polyneuropathy (CIDP), myasthenia gravis (MG), and several other rare diseases such as dermatomyositis (DM) [1,2]. Additionally, IVIG is currently under evaluation for many other diseases (e.g., rheumatoid arthritis) and is often prescribed off label [3].

Several mechanisms of action have been proposed for the anti-inflammatory effect of high-dose IVIG. Some of these are dependent on the F(ab’)_2_ portion and include neutralization of autoAbs by anti-idiotypic Abs and binding/neutralization of immune mediators, such as cytokines. Other protective mechanisms are mediated by the Fc domain and include the blockade of Fc gamma receptors (FcγRs), saturation of the neonatal FcR (FcRn) to enhance autoAb clearance, scavenging of complement protein fragments, modulation of immune cell activity (regulatory T cells, B cells or tolerogenic dendritic cells), or up-regulation of the inhibitory FcγRIIB (CD32B) [4]. A therapeutic role for the IgG Fc domain in autoimmune diseases has been suggested in experimental models of arthritis and ITP [5]. Furthermore, in a clinical study plasma-derived monomeric Fc successfully ameliorated acute ITP in children [6]. While these mechanisms can be achieved with IVIG, its production is dependent on the supply of human plasma and high doses are required for immunomodulation (1000–2000 mg/kg body weight), which remain challenges for this product. The availability of a recombinant alternative that can reproduce key mechanisms of action at lower doses is therefore desirable.

Several recent studies have investigated the in vivo efficacy of recombinant Fc (recFc) protein-based therapeutics. Various approaches for controlled multimerization of Fc to form polyvalent molecules have been explored [1,7]. Fusion of the human IgG2 hinge region to human IgG1 Fc or mouse IgG2a Fc led to the expression of multimerized Fc fragments that bound FcγR with high avidity [8]. These molecules demonstrated therapeutic efficacy in animal models of arthritis and ITP [8], as well as in a model of inflammatory neuropathy [9] and in experimental autoimmune MG [10]. Interestingly, in most models, efficacy was achieved with low doses of approximately 50 mg/kg body weight, compared to the standard dose of 1000–2000 mg/kg for IVIG in inflammatory indications. Using an alternative strategy, Ortiz et al. [11] studied Fc multimers of increasing valency and identified molecules that bound FcγRs with high avidity without triggering activating signals. A trivalent molecule termed CSL730 (earlier named Fc3Y) showed protection in mouse models of ITP, arthritis, and epidermolysis bullosa acquisita at doses as low as 25 mg/kg [11]. Finally, a hexameric Fc molecule with increased binding to FcγRs and DC-SIGN (CD209) appeared to fully activate complement (C1q binding and C5b-9 deposition) [12]. In addition, two other similar human IgG1-Fc hexamers were reported not to induce downstream activation of complement but promote cleavage of C4 [13,14]. Neither of these molecules has progressed into human trials, and their protective mechanisms of action have not been fully elucidated in human disease conditions. In this study, we developed recFc with mutations and different valencies to increase the affinity to C1q and thereby enhance their potency to prevent auto-Ab-mediated classical pathway activation. In addition, to optimize the pharmacokinetic properties of the dual inhibitors, the R292P mutation was selected based on previous published studies [15,16] showing the importance of this mutation for clearance of multivalent IgG, such as in small immune complexes. These studies indicate that FcγRIIB plays a key role in the removal of small immune complexes from circulation in mice and rats. FcγRIIB is highly expressed in liver sinusoidal endothelial cells (LSECs) in mice, rats, and humans [17].

## 2. Results

### 2.1. Generation of Mono-, Di- and Trivalent Fc Molecules with Increased Binding Affinity to Complement Protein C1q

To investigate strategies for enhancing inhibition of the classical complement pathway, we generated recombinant human IgG1-Fc molecules carrying point mutations designed to increase the binding affinity to complement component C1q which, together with serine proteases C1s and C1r, forms part of the C1-complex. In addition to enhancing binding affinity, the hIgG1-Fc valency was increased from mono- to di- to trivalent molecules (termed Fc1, Fc2 and Fc3Y, respectively, see Table 1).

### 2.2. Binding Strength of Mono-, Di- and Trivalent Fc Molecules to C1q Is Enhanced Exponentially via Affinity and Avidity

**Figure 1:**
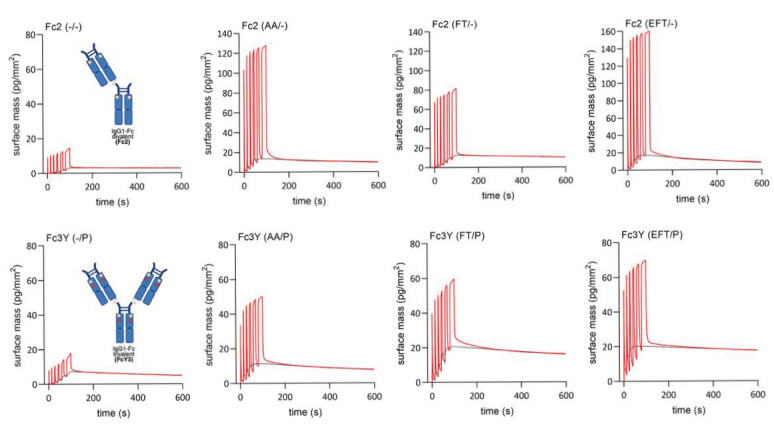
Representative sensorgrams of di- (Fc2) and trivalent (Fc3Y) hIgG1-Fc binding to C1q. Measurements were performed using the RAPID kinetics method in a Creoptix WAVE biosensor with biotinylated C1q immobilized onto a Streptavidin-coated surface, and Fc molecules pulse-injected as analyte. Measured data (in red) were cropped at the beginning (50–100 s) of the dissociation phase to allow fitting of a 1:1 Langmuir model (superimposed grey lines).

The introduction of C1q-directed mutations (referred to as AA, FT and EFT) in bivalent recombinant Fc variants (Fc2), designed to enhance intrinsic binding affinity, produced a modest increase in apparent affinity (K_D_) to C1q. This effect was potentiated when the hIgG1-Fc was converted to a trimeric form (Fc3Y), where increased valency enhanced avidity and strengthened binding (see Table 2 and Figure 1). Collectively, these findings underscore the synergistic interplay between intrinsic affinity and multivalent avidity when optimizing Fc-C1q interactions.

### 2.3. Mutant recFc Molecules Inhibit Classical Pathway Activation Without C4a Generation

**Figure 2:**
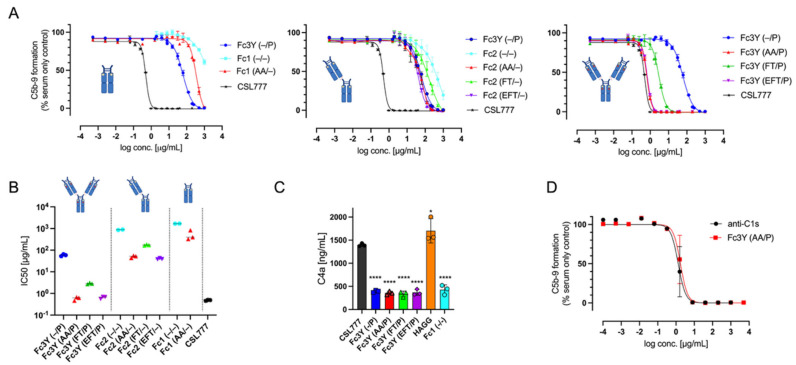
Complement inhibition, activation, and benchmarking against hexavalent hIgG1-Fc (CSL777). (**A**) Inhibition of complement by various recombinant hIgG1-Fc molecules by WIESLAB ELISA CP. Data show means ± SD of three individual experiments. (**B**) IC50 values calculated for all measured hIgG1-Fc molecules. (**C**) Complement activation in normal human serum by CSL777, Fc3Y (-/P), Fc3Y (AA/P), Fc3Y (FT/P), and Fc3Y (EFT/P) at 62.5 μg/mL was measured by C4a generation in normal human serum by ELISA. Data show means ± SD of three individual experiments. * *p* < 0.05, **** *p* < 0.0001, one-way ANOVA with Dunnett test, compared to the benchmarking molecule CSL777, positive control heat-aggregated IgG (HAGG) and negative control Fc1 (-/-), both at 1 mg/mL. (**D**) Benchmark study of Fc3Y (AA/P) against anti-C1s mAb. Data show means ± SD of two individual experiments.

Mutations that improved C1q interaction (referred to as AA, FT and EFT) conferred a marked increase in inhibitory potency compared to wild-type IgG1-Fc (Figure 2A). Importantly, the improvement was not solely explained by higher binding affinity to C1q. When Fc constructs were multimerized to increase valency from monovalent to divalent and trivalent configurations, the inhibitory effect was amplified exponentially (Figure 2B) (e.g., IC50 values: monovalent Fc1 (AA/-) vs. Fc2 (AA/-) vs. Fc3Y (AA/P): 517.0 vs. 50.5 vs. 0.6 μg/mL; see Supplemental Table S1). Trivalent Fc molecules exhibited the most pronounced inhibitory effect, achieving near-complete blockade of classical pathway activation at substantially lower concentrations than the monovalent counterpart. In particular, the variants Fc3Y (AA/P) and Fc3Y (EFT/P) demonstrated higher potency than the variant Fc3Y (FT/P) (IC50: 0.6 vs. 0.6 vs. 2.9 μg/mL, respectively, see Supplemental Table S1). The R292P mutation had no impact on the potency to inhibit the classical pathway (see Supplemental Figure S2). In contrast to CSL777, which inhibits the classical pathway but still results in C4a generation, none of the trimers (mutated or not) showed increased C4a (Figure 2C). Overall, the level of classical pathway inhibition achieved with the C1 mutant trimer was comparable to that seen with a specific anti-C1s mAb (Figure 2D).

### 2.4. Assessment of How C1q-Enhancing Mutations in Fc Molecules Affect Binding to Fcγ Receptors, Measured by SPR, and Phagocytosis in THP-1 Cells

**Figure 3:**
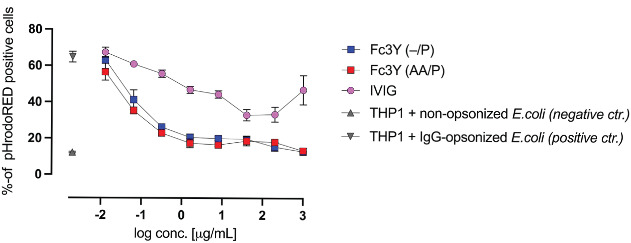
Inhibition of antibody-dependent cellular phagocytosis of IgG-opsonized *E. coli* pHrodo™ Red bioparticles by THP1 cells. IVIG was used as control. Results from a single experiment are shown as mean ± SEM of technical duplicates (of n = 2 individual performed experiments).

Introduction of C1q-targeting mutations did not measurably affect interactions of Fc3Y (AA/P) and Fc3Y (FT/P) with Fcγ receptors. Surface plasmon resonance (SPR) analysis demonstrated comparable steady state binding and kinetics for variants Fc3Y (AA/P) and Fc3Y (FT/P) binding to FcγRI (CD64), FcγRIIA and B (CD32A and B), and FcγRIIIA (CD16A); compared to Fc3Y (-/-), both variants showed reduced binding to FcγRIIA/B but similar binding to FcγRIIIA and FcγRI (Table 3 and Supplemental Figure S1). For Fc3Y (EFT/P), binding to FcγRIIA/B increased, showing that EFT mutations likely offset the R292P mutation. However, the C1q mutations introduced in the molecule Fc3Y (EFT/P) showed a decrease in binding affinity to FcγRIIIA F158 (Table 3). Consistent with these biophysical data, functional assessment in a THP-1-based phagocytosis assay revealed no differences in FcγR-mediated effector functions for Fc3Y (AA/P) compared to Fc3Y (-/P), exhibiting similar levels of inhibition of cellular uptake (Figure 3). The mutation R292P decreased in particular binding to FcγRII (Supplemental Table S2). Together, these results indicate that the engineered mutations in Fc3Y (AA/P) selectively modulate C1q binding while maintaining antagonistic FcγRIIA receptor interactions and downstream effector function.

### 2.5. Reducing Binding Affinity to FcγRIIB (CD32B) Increases Half-Life of recFc Constructs

**Figure 4:**
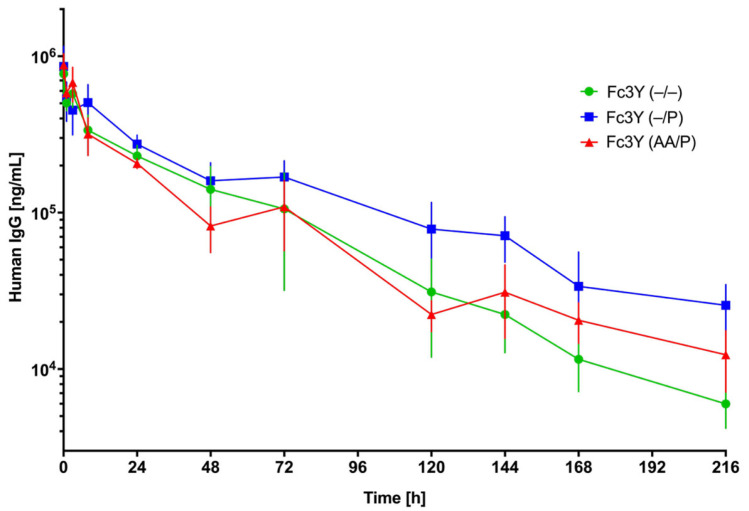
Pharmacokinetic profiles of plasma IgG1 after administration of Fc3Y (-/-), Fc3Y (-/P), and Fc3Y (AA/P) at a dose of 25 mg/kg IV to hFcRn Tg mice (n = 4 per timepoint, mean ± SD). Detection limit: 1560 ng/mL.

Previous studies have shown that recFc multimers can be rapidly cleared from the body via their binding to FcγRIIB on liver sinusoidal endothelial cells (LSECs) [16]. Based on the potent inhibition of the classical pathway and preserved binding to FcγRs, the trivalent molecule with enhanced affinity binding to C1q, Fc3Y (AA/P), was selected for PK characterization in vivo in comparison to trivalent variants with normal C1q affinity. Further, the potential to enhance the half-life of recFc molecules by reducing the interaction with the FcγRIIB receptor was investigated (designated (P)). For this purpose, the PK profile of three trivalent Fc molecules was evaluated in human FcRn Tg mice, demonstrating that modifications decreasing FcγRIIB binding resulted in a prolonged plasma half-life for the trivalent Fc molecule (Figure 4 and Table 4; Fc3Y (-/P) t_1/2_ = 49 h and Fc3Y (AA/P) t_1/2_ = 56 h vs. Fc3Y (-/-) t_1/2_ = 35 h). Please note that the group treated with Fc3Y (AA/P) had relevant differences in plasma exposure between the animal cohorts, leading to a zig-zag curve; nevertheless, the overall interpretation of data was still possible, and non-compartmental analysis remained within quality acceptance criteria.

In line with the observed t_1/2_ differences, the area under the plasma concentration curve until the last timepoint (AUC_last_) was higher for Fc3Y (-/P) compared to Fc3Y (-/-) (Fc3Y (-/P) AUC_last_ = 29,857 h*µg/mL vs. Fc3Y (-/-) AUC_last_ = 20,711 h*µg/mL). In contrast, AUC_last_ was comparable between the Fc3Y (-/-) and Fc3Y (AA/P) groups. This could be explained by two effects cancelling each other out: while terminal t_1/2_ was higher for Fc3Y (AA/P) than Fc3Y (-/-) related to FcγRIIB binding, exposure of Fc3Y (AA/P) dropped quicker in the initial phase (Fc3Y (AA/P) = −460 µg/mL vs. Fc3Y (-/-) = −288 µg/mL), which may be related to quick target binding to C1q. Overall, the initial distribution was stronger for Fc3Y (AA/P) compared to Fc3Y (-/-) and Fc3Y (-/P), while drug elimination in the terminal phase was comparable between Fc3Y (AA/P) and Fc3Y (-/P) and lower than Fc3Y (-/-). This indicates that, while enhancing C1q affinity can impact pharmacokinetics in the initial distribution phase, the extension of plasma half-life achieved by reducing FcγRIIB binding remains beneficial.

## 3. Materials and Methods

### 3.1. Generation of Constructs and Transient Expression in Expi293 Cells

Sequences were obtained from Momenta, and plasmids separately encoding long and short chains were directionally cloned into pcDNA3.1(neo) (Thermo Fisher Scientific, Waltham, MA, USA). The long chain encodes two tandem Fc sequences joined by a 20-amino acid glycine linker. The short chain encodes a single Fc sequence. To favour assembly of the intact molecule, knobs-into-holes and electrostatic steering mutations were introduced. Additionally, an engineered disulphide linkage was introduced by engineering cysteine residues in the Fc2 and Fc3 CH3 domains. Variant sequences were also generated: a double (K326A, E333A) mutation was introduced to increase C1q binding to enhance potency and efficacy in blocking complement-mediated activities [18]. Alternatively, the H268F and S324T, or S267E, H268F, and S324T mutations were introduced to achieve this [19]. (See amino acid sequences in the Supplemental Data) All mutations were generated using standard techniques. Fc molecules were produced in the transient transfection using the Expi293™ Expression System (Thermo Fisher Scientific) according to the manufacturer’s instructions, and as described recently [20]. Plasmids encoding the long and short chains were co-transfected to form the intact molecule. RecFc preparations were purified using standard Protein A affinity purification techniques. Initial affinity capture of Fc molecules was performed using routine Protein A chromatography. The dimeric and trimeric species were further purified using an SCX column, POROS XS, binding in 50 mM MES, pH 6.0 and eluting with 50 mM MES, pH 6.0 + 400 mM NaCl using a linear gradient 0–50% B over 50 CV. The desired species was pooled and either buffer-exchanged into PBS pH 7.4 using a HiPrep 26/10 column or further purified using a Superdex 200 gel filtration column. The resulting material was analyzed by non-reducing and reducing SDS-PAGE as well as analytical sizing, showing highly purified proteins (>95% purity). The anti-C1s mAb (“Riliprubart-like”) was generated according to the AA-sequence in patent WO201807676A1. The recombinant molecules used in this study were shown to be endotoxin-free by the LAL-test.

### 3.2. Binding of Fc Constructs to Complement Protein C1q by WAVE

Binding kinetics analysis was performed on a Creoptix WAVE GCI biosensor (Malvern Panalytical, Westborough, MA, USA) using streptavidin-coated sensor chips (WAVEchip 4PCP-STA). All experiments were performed with PBS supplemented with 1% BSA and 0.005% Tween-20 as running buffer at 25 °C. Biotinylated C1q was immobilized onto the sensor surface via streptavidin coupling, achieving a surface density of approximately 450 pg/mm^2^ on the measurement channels. No immobilization was conducted on the reference channel.

Kinetic measurements were carried out using the waveRAPID (Repeated Analyte Pulses of Increasing Duration) assay format at 25 °C [21]. Analyte solutions at 1–10 µM, prepared in running buffer, were pulse-injected for 100 s over the measurement and reference channels at a flow rate of 50 μL/min per channel. Sensorgrams were processed using the WAVEcontrol software v4.7.4 (Malvern Panalytical) following a double-reference subtraction strategy, and the first 50–100 s of dissociation were cropped and excluded from analysis. Data were fit with a one-to-one Langmuir model to provide an estimate for the kinetic constants. All experiments were performed in at least duplicate.

### 3.3. Binding of Fc Constructs to FcγRs by SPR

Affinities were measured using a Biacore^®^ 8K+ SPR Biosensor (Cytiva, Marlborough, MA, USA) on a Protein G Series S CM5 sensor chip (Cytiva). Fc molecules were captured on active flow cells at the beginning of each cycle to a surface density of ~600 response units. Flow cell 2, in which no Fc constructs were captured, was used as a reference. Recombinant Fcγ receptors FcγRIIIA F158 (CD16A), FcγRIIA R131 (CD32A), and FcγRIIB (CD32B) were injected for 60 s at concentrations between 390.6 nM and 50 µM (made in a 2-fold dilution series), and a dissociation of 120 s was allowed. Recombinant FcγRI (CD64) receptor was injected for 300 s at concentrations between 0.078 and 10 nM (made in a 2-fold dilution series), and a dissociation of 900 s was monitored. All Fcγ receptor concentrations were injected in duplicate over both flow cells, and buffer blanks were included for referencing purposes. After each cycle, the surface was regenerated with a 10 mM glycine pH 1.6; 60 s. The analysis was performed at 37 °C at a flow rate of 10 µL/min for steady state interactions and 30 µL/min for kinetic interactions. The experiment was conducted in 10 mM HEPES, 150 mM NaCl, 3 mM EDTA, 0.005% *v/v* surfactant P20 adjusted to pH 7.4. Biacore Insight Evaluation software 5.0.18 (Cytiva) was used to fit double-referenced sensorgrams to a 1:1 steady state with a global R_max_ value, and to a 1:1 kinetics model including a term for mass transport limitation. The R_max_ value for kinetic fitting was fitted locally to account for slight deviations in the level of Fc constructs captured, while the association rate (*k*_a_), dissociation rate (*k*_d_), and equilibrium (*K*_D_) were fitted globally.

### 3.4. Effect of Fc Constructs on Complement Deposition (WIESLAB ELISA)

The effect of Fc constructs on the function of the classical complement pathway was examined in the Wieslab^®^ Complement System Screen (Euro-Diagnostica, Malmø, Sweden), which is an enzyme immunoassay for the specific detection of the three pathways, with deposition of C5b-9 (detected with an anti C9 neo-epitope mAb) as a common read-out. Samples were preincubated with 1 or 20% NHS (indicated in the figure legend) diluted with GVB^2+^ buffer as described above. After 1 h incubation at 37 °C, the samples were transferred to the classical pathway ELISA plates. Further dilutions were made according to the instructions, i.e., 1:100 for the CP. Recombinant hIgG1-Fc hexamer (CSL777) was used as a control [13].

### 3.5. Generation of C4a in Normal Human Serum (NHS)

For the assay, normal human serum (NHS) was diluted 1:5 with GVB2+ buffer (Complement Tech, Tyler, TX, USA) and incubated for 1 h at 37 °C as described in Spirig et al. [13]. The reaction was stopped by adding EDTA. Activation of human complement in serum was analyzed by the generation of C4a by ELISA (Quidel, San Diego, CA, USA).

### 3.6. THP1 Cell Lines and Culture

The human monocytic cell line, THP1 (ATCC), was cultured in RPMI 1640 medium containing 10% FCS, and 1% (100 U/mL) penicillin/streptomycin. Cells were passaged every 3–4 days and culture medium was replaced.

### 3.7. Phagocytosis Assay with THP-1 Cells

THP-1 cells were pre-incubated with indicated hIgG1-Fc molecules for 45 min on ice, followed by incubation with IgG-opsonized *E. coli* pHrodo™ Red bioparticles (Thermofisher Scientific, Waltham, MA, USA) for 3 h in the presence of IVIG, Fc3Y (-/P), or Fc3Y (AA/P) at 37 °C. The pHrodo™ Red bioparticles conjugates for phagocytosis are non-fluorescent outside the cell at neutral pH but fluoresce brightly in acidic pH environments, such as those of endosomes and lysosomes. Afterwards, uptake of beads was analyzed by flow cytometry. For the control, non-IgG-opsonized *E. coli* pHrodo™ bioparticles were used.

### 3.8. PK Study in hFcRn Tg Mice

Mouse studies were performed at the Department of Pharmacology and Toxicology, CSL Behring GmbH, Marburg, Germany. All animals were handled and managed in accordance with animal care protection laws. Ethics approval for the mouse studies was obtained from Regierungspraesidium Giessen, Wetzlar, Germany (approval number A5/2020). Male C57BL/6J human FcRn homozygous transgenic mice (FcRn Tg; line 32; designated as 32HOM) were obtained from Charles River Laboratories, Erkrath, Germany, and used in a body weight range of 30–50 g. Fc3Y (-/-), Fc3Y (-/P), or Fc3Y (AA/P) were administered intravenously at a dose of 25 mg/kg. The animals were separated into two cohorts per treatment group (n = 4 animals per timepoint), and blood samples were drawn between 5 min and 216 h post-dose from the saphenous vein (0.083 h (n = 8 mice), 1 h, 3 h, 8 h, 24 h, 48 h, 72 h, 120 h, 144 h, 168 h, 216 h).

Blood samples were mixed with 10% EDTA and then processed to EDTA plasma and stored at −70 °C. Analysis of recFc constructs in plasma samples of mice administered with these agents was performed using a human IgG enzyme-linked immunosorbent assay (ELISA). Capture antibody was goat anti-human IgG Fc (Sigma, Cat. No. I2136, Germany; RRID:AB_260147); blocking solution was BSA-based (Sigma Aldrich, Schnelldorf, Germany); detection antibody was goat anti-human IgG Fc conjugated to horseradish peroxidase (HRP; Sigma Aldrich, Schnelldorf, Germany).

PK analysis was performed by non-compartmental analysis using Phoenix WinNonLin 8.3, applying the sparse function and linear trapezoidal linear interpolation with uniform weighting.

### 3.9. Experimental Design and Analysis

Group numbers (n) and analysis approaches were predetermined before initiation of experiments.

### 3.10. Software

GraphPad Prism 11.0.0 was used for graphing. Phoenix WinNonLin 8.3 was used for non-compartmental analysis. Analyses of differences between sample groups were performed using the tests indicated in the text. Data shown are means ± SD, unless otherwise stated. A *p* value < 0.05 was considered statistically significant.

## 4. Discussion

In this study, we demonstrated that recombinant human IgG1-Fc molecules engineered for increased valency and enhanced C1q affinity can achieve potent inhibition of the classical complement pathway, while simultaneously antagonizing Fcγ receptor-mediated effector functions. Our results showed that increasing the valency of the Fc domain, particularly in the trivalent format, led to a marked gain in functional potency for complement inhibition. This effect was further amplified by the introduction of targeted C1q-binding mutations, as evidenced by the exponential increase in inhibitory activity observed in both biochemical and cellular assays. The trivalent Fc3Y (AA/P) molecule in particular displayed sub-micromolar IC50 values in Wieslab ELISA assays, outperforming both monomeric and dimeric Fc constructs as well as benchmarked hexavalent Fc molecules. A key finding of our work was that the engineered trivalent Fc3Y (AA/P) acted as a highly effective C1q decoy, sequestering C1q in solution and thereby preventing its deposition and activation on immune complexes. Unlike previously described multimeric Fc constructs, such as hexamers or other Fc multimers (e.g., GL-2045), which have been associated with unintended complement activation and C4a generation, our trivalent molecule did not induce C4a release in normal human serum. In addition to initial complement activation, it has been demonstrated that these Fc multimers generated by fusion of the human IgG2 hinge region to human IgG1 Fc also induced activation of Nuclear Factor of Activated T cells (NFAT) in human FcγRIIA- and FcγRIIIA-expressing cells [22]. This suggests a mechanism of action that is fundamentally distinct from complement activation, instead relying on competitive inhibition at the level of C1q engagement. An earlier study comparing FT vs. EFT mutations in the Fc of full-length recombinant IgG1 demonstrated that the serine replaced by glutamic acid in EFT increased binding affinity to C1q, which correlated with increased potency in functional complement-dependent cytotoxicity assays [19]. The ability of Fc3Y (AA/P) to inhibit C1q further supports its role as a selective inhibitor of the classical pathway. In addition to its complement inhibitory properties, the trivalent Fc3Y (AA/P) retained high-avidity binding to Fcγ receptors and effectively blocked FcγRIIa-mediated effector functions, such as antibody-dependent cellular phagocytosis in THP-1 cells. This dual antagonism is particularly relevant in autoimmune and inflammatory diseases where both complement-mediated tissue injury and FcγR-driven inflammation contribute to pathology.

Pharmacokinetic studies in hFcRn transgenic mice revealed—even though increased variability was observed related to animal cohort effects—that reducing the binding affinity of the trivalent Fc construct to FcγRIIB resulted in an extension of terminal plasma half-life. This addresses a key limitation of earlier multimeric Fc constructs, which were rapidly cleared due to strong FcγRIIB binding. In addition, the impact of FT and EFT on FcγRI-III binding in full-length IgG1 has shown increased binding to human FcγRIIB by SPR, but not to cynomolgus FcγRIIB [23]. The potential prolonged terminal t_1/2_ of Fc3Y (AA/P) as compared to Fc3Y (-/-) with normal FcγRIIB binding might offer a practical advantage for clinical translation, but further studies in rodent, non-rodent, and humans are needed to confirm the potential of R292P for extending the half-life of multimeric hIgG1-Fc molecules. Importantly, the absence of C4a generation and the selective inhibition of the classical pathway suggest a favourable safety profile for the trivalent Fc3Y (AA/P) molecule compared to hexameric [13,14] or multimeric hIgG1-Fc molecules [24,25].

Several limitations of this study should be acknowledged. First, the potential improved pharmacokinetic properties associated with the R292P mutation [16] was demonstrated only in human FcRn transgenic mice, limiting the translational relevance to humans and other non-rodent species. Second, SPR analyses of FcγR and C1q binding were performed only using human proteins, without cross-species characterization in rodent or non-rodent systems, despite known species-specific differences in Fc biology. Finally, the dataset supporting FcγR effector function inhibition by the dual-acting molecules remains limited, as functional assessment was largely restricted to receptor binding and a THP-1 phagocytosis assay, without broader evaluation of FcγR-mediated immune functions. An important consideration for the trivalent human IgG1-Fc molecules is their interaction with the inhibitory FcγRIIB. While transient blockade or reduced engagement of FcγRIIB may improve systemic exposure and enhance therapeutic potency by preventing rapid clearance via liver sinusoidal endothelial cells [16], prolonged interference with this inhibitory pathway could also affect immune homeostasis. As FcγRIIB plays a central role in limiting immune cell activation and maintaining peripheral tolerance [26], excessive or sustained blockade may theoretically increase the risk of unwanted immune activation or inflammatory responses. Therefore, achieving an optimal balance between improved pharmacokinetics, therapeutic efficacy, and preservation of FcγRIIB-mediated inhibitory signalling will be important for the safety profile of these engineered Fc molecules.

Collectively, our findings establish trivalent, C1q-affinity-enhanced human IgG1-Fc molecules as potent, dual-function inhibitors of both complement and FcγR pathways. These constructs recapitulate and surpass key anti-inflammatory mechanisms of IVIG at substantially lower doses, with improved pharmacokinetics and safety. Future studies will focus on in vivo efficacy in disease models, detailed mechanistic dissection of complement and FcγR blockade, and translational development towards clinical application in autoimmune and inflammatory diseases.

## Figures and Tables

**Table 1:** Overview of mono- (Fc1), di- (Fc2), and trivalent (Fc3Y) human IgG1-Fc molecules containing different mutations to increase affinity to C1q and reduce binding to FcγRIIB (only for trivalent molecules). All proteins were formulated in PBS. N/A: no mutations are inserted to increase affinity binding.

Molecule ID	C1q Mutations	FcγRIIB Mutation
**Trivalent**		
Fc3Y (-/-)	N/A	N/A
Fc3Y (-/P)	N/A	R292P (P)
Fc3Y (AA/P)	K326A/E333A (AA)	R292P (P)
Fc3Y (FT/P)	H268F/S324T (FT)	R292P (P)
Fc3Y (EFT/P)	S267E/H268F/S324T (EFT)	R292P (P)
**Divalent**		
Fc2 (-/-)	N/A	N/A
Fc2 (AA/-)	K326A/E333A (AA)	N/A
Fc2 (FT/-)	H268F/S324T (FT)	N/A
Fc2 (EFT/-)	S267E/H268F/S324T (EFT)	N/A
**Monovalent**		
Fc1 (-/-)	N/A	N/A
Fc1 (AA/-)	K326A/E333A (AA)	N/A

**Table 2:** Binding kinetics of hIgG1-Fc molecules to C1q measured by GCI. Values for k_a_ and K_D_ were estimated based on fitting of a 1:1 model to the slowest dissociation phase. N/A: not available (interaction is very weak).

Molecule	n	K_D_ [nM]	k_a_ (M^−1^s^−1^)	k_d_ (s^−1^)
Fc3Y (-/P)	3	20.45 ± 1.16	3.66 ± 0.16 × 10^4^	7.49 ± 0.75 × 10^−4^
Fc3Y (AA/P)	3	3.01 ± 0.66	3.04 ± 0.16 × 10^5^	9.13 ± 1.71 × 10^−4^
Fc3Y (FT/P)	3	3.04 ± 1.02	1.99 ± 0.27 × 10^5^	6.18 ± 2.86 × 10^−4^
Fc3Y (EFT/P)	3	0.34 ± 0.02	7.63 ± 0.51 × 10^5^	2.47 ± 0.26 × 10^−4^
Fc2 (-/-)	2	86.97 ± 11.71	2.91 ± 0.62 × 10^3^	2.50 ± 0.28 × 10^−4^
Fc2 (AA/-)	3	34.84 ± 6.01	3.54 ± 0.43 × 10^4^	1.26 ± 0.56 × 10^−3^
Fc2 (FT/-)	3	49.64 ± 15.25	1.09 ± 0.72 × 10^4^	4.66 ± 1.30 × 10^−4^
Fc2 (EFT/-)	3	37.93 ± 4.94	4.19 ± 0.60 × 10^4^	1.58 ± 0.35 × 10^−3^
Fc1 (-/-)	2	N/A	N/A	N/A
Fc1 (AA/-)	2	N/A	N/A	N/A

**Table 3:** Binding affinities of hIgG1-Fc molecules to FcγR measured by SPR (Biacore 8k+). Affinity (K_D_) values were derived by fitting binding profiles to a 1:1 steady state model for weak/fast interactions and 1:1 kinetic model for strong interactions.

	FcγRIIA R131 (n = 2)	FcγRIIB (n = 4)	FcγRIIIA F158 (n = 4)	FcγRI (n = 4)
Molecule	K_D_ [nM], Steady State Affinity			K_D_ [pM], Kinetics
Fc3Y (-/-)	1330 ± 1	3280 ± 64	4990 ± 8.2	79.7 ± 0.1
Fc3Y (AA/P)	3040 ± 2.8 (~0.4×)	7680 ± 32 (~0.4×)	5850 ± 12.3 (~0.9×)	95.3 ± 0.03 (~0.8×)
Fc3Y (FT/P)	3550 ± 16 (~0.4×)	7980 ± 125 (~0.4×)	9550 ± 112 (~0.5×)	74.6 ± 0.03 (~1.1×)
Fc3Y (EFT/P)	524 ± 1.7 (~2.5×)	1270 ± 3 (~2.6×)	22,400 ± 1380 (~0.2×)	50.5 ± 0.07 (~1.6×)
Fc1 (AA/-)	983 ± 3.8 (~0.7×)	2410 ± 17 (~1.4×)	3990 ± 16 (~1.3×)	80.6 ± 0.05 (~1.0×)
Fc1 (-/-)	1460 ± 2.2 (~0.9×)	3280 ± 64 (~1.0×)	7610 ± 24.3 (~0.7×)	86.2 ± 0.03 (~0.9×)

**Table 4:** Pharmacokinetic parameters from non-compartmental analysis of Fc3Y (-/-), Fc3Y (-/P), and Fc3Y (AA/P) administered at a dose of 25 mg/kg IV to hFcRn Tg mice (n = 4 per timepoint). Exposure drop was calculated as percent difference ([value-baseline]/baseline). Data are presented as mean and SD (exposure drop) or SE (C_max_, AUC_last_). AUC_last_: area under plasma concentration curve until last 9 days post dose; MRT: mean residence time.

Study Group	C_max_ [µg/mL]	Exposure Drop in First 8 h [µg/mL]	AUC_last_ [h*μg/mL]	Terminal t_½_ ([h])
Fc3Y (-/-)	773 ± 71	−288 ± 105	20,711 ± 1877	35
Fc3Y (-/P)	777 ± 128	−298 ± 232	29,857 ± 2283	49
Fc3Y (AA/P)	880 ± 56	−460 ± 134	20,627 ± 1362	56

## Data Availability

The original contributions presented in this study are included in the article/Supplementary Materials. Further inquiries can be directed to the corresponding authors.

## Supplementary Materials

The following supporting information can be downloaded at: https://www.mdpi.com/article/10.3390/cells15131156/s1, Figure S1. Representative sensorgrams of Fc3Y (AA/P) hIgG1-Fc molecule binding to Fcγ receptors: IIA, IIB/C, IIIA and I. Measurements were performed in a capture format, where the Fc molecules were captured via Protein G and Fcγ receptors injected as analytes. Measured data (black fitted curves) were fitted to colored experimental curves using a 1:1 Steady State model or a Kinetic Langmuir model to derive the strength of interactions (KD); Supplemental Table S1: IC50 values for hIgG1-Fc mediated inhibition of the classical complement pathway. Supplemental Table S2: Binding affinities of Fc3Y (-/-) vs Fc3Y (-/P) molecule to Fc3R measured by SPR (Biacore A100 enhanced). Affinity (KD) values were derived by fitting binding profiles to a 1:1 Steady State model for weak/fast interactions and 1:1 Kinetic model for strong interactions. Figure S2: (A) Inhibition of complement dependent cytotoxicity (CDC) by anti-AQP4 IgG1 mAb opsonized AQP4 expressing CHO cell line by CSL777, Fc3Y (-/-) and Fc3Y (-/P). Data show the percentage to NHS values (mean ± SD, n = 5–6). (B) Inhibition of C3b deposition on HUVEC by CSL777, Fc3Y (-/-), and Fc3Y (-/P). Data show the percentage of C3b normalized to NHS values (mean ± SD, n = 3). References [13,27] are cited in Supplementary Materials.
