# Supplementary material for: Engineered Trivalent Human IgG1-Fc Proteins for Potent Complement Inhibition
Source: Cells. 2026 Jun 25;15(13):1156. doi: 10.3390/cells15131156 (PMC13360016; doi:10.3390/cells15131156)
Supplement: Supplementary file 1 [file cells-15-01156-s001.zip › cells-4323217-supplementary.pdf]

Supplementary Data Campbell *et al* 2026

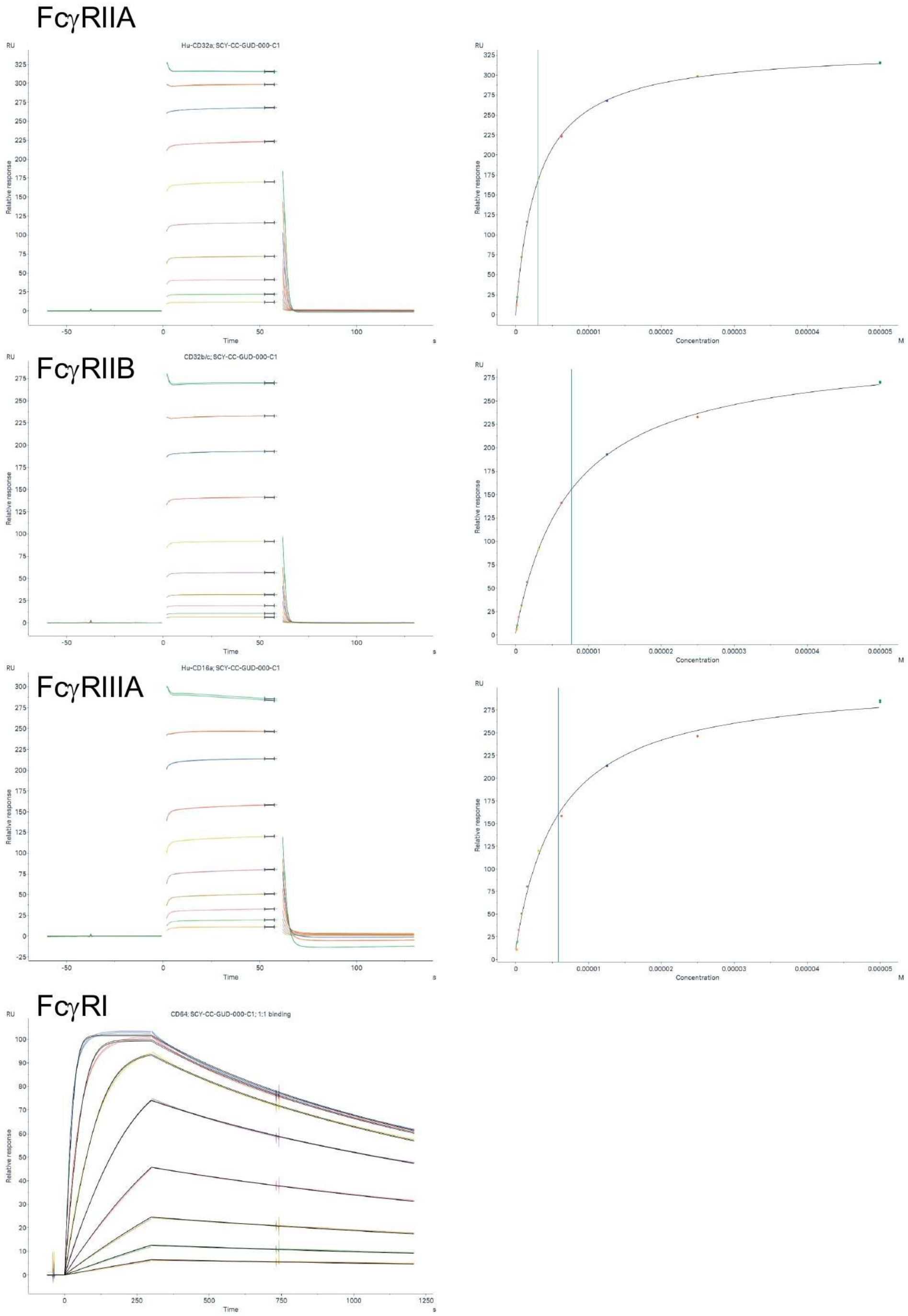

Supplemental Figure S1: Representative sensorgrams of Fc3Y (AA/P) hIgG1-Fc molecule binding to Fcγ receptors: IIA, IIB/C, IIIA and I. Measurements were performed in a capture format, where the Fc molecules were captured via Protein G and Fcγ receptors injected as analytes. Measured data (black fitted curves) were fitted to colored experimental curves using a 1:1 Steady State model or a Kinetic Langmuir model to derive the strength of interactions (K_D).

Molecule	n	IC50 (mean ± SD)
Fc3Y (-/P)	3	58.7 ± 5.7
Fc3Y (AA/P)	3	0.6 ± 0.1
Fc3Y (FT/P)	3	2.9 ± 0.2
Fc3Y (EFT/P)	3	0.6 ± 0.1
Fc2 (-/-)	2	876.4 ± 31.6
Fc2 (AA/-)	3	50.5 ± 6.3
Fc2 (FT/-)	3	173.8 ± 11.3
Fc2 (EFT/-)	3	39.6 ± 2.7
Fc1 (-/-)	2	1677.0 ± 15.5
Fc1 (AA/-)	3	517.0 ± 271.0
CSL777	3	0.5 ± 0

Supplemental Table S1: IC50 values for hIgG1-Fc mediated inhibition of the classical complement pathway.

	**FcγRIIA R131** (n=3)	**FcγRIIB** (n=3)	**FcγRIIIA F158** (n=3)	**FcγRI** (n=2)
**Molecule**	K_D [nM ± SEM], Steady State Affinity			K_D [pM], Kinetics
Fc3Y (-/-)	690 ± 70	2000 ± 20	2200 ± 100	135
Fc3Y (-/P)	2900 ± 50 (~ 0.2x)	9100 ± 10 (~ 0.2x)	4400 ± 220 (~ 0.5x)	147.5

Supplemental Table S2: Binding affinities of Fc3Y (-/-) vs Fc3Y (-/P) molecule to FcγR measured by SPR (Biacore A100 enhanced). Affinity (K_D) values were derived by fitting binding profiles to a 1:1 Steady State model for weak/fast interactions and 1:1 Kinetic model for strong interactions.

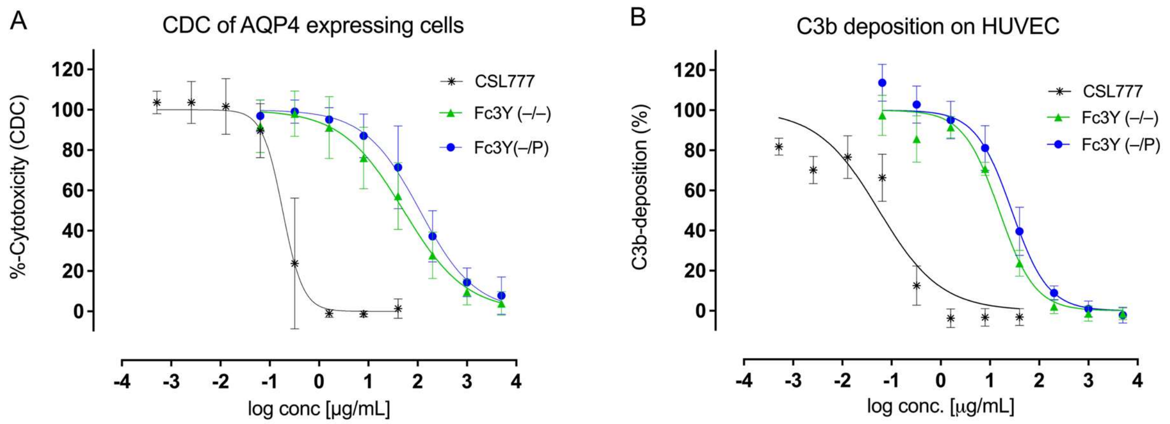

Supplemental Figure S2: A) Inhibition of complement dependent cytotoxicity (CDC) by anti-AQP4 IgG1 mAb opsonized AQP4 expressing CHO cell line by CSL777, Fc3Y (-/-) and Fc3Y (-/P). Data show the percentage to NHS values (mean ± SD, n = 5-6). B) Inhibition of C3b deposition on HUVEC CSL777, Fc3Y (-/-), and Fc3Y (-/P). Data show the percentage of C3b normalized to NHS values (mean ± SD, n = 3).

Amino Acid Sequence of generated molecules

Fc3Y (-/-)

Long chain (knob and holes sequences in blue, electrostatic steering mutations in purple)

DKTHTCPPCPAPELLGGPSVFLFPPKPKDTLMISRTPEVTCVVVDVSHEDPEVKFNWYVDGVEV
HNAKTKPREEQYNSTYRVVSVLTVLHQDWLNGKEYKCKVSNKALPAPIEKTISKAKGQPREPQ
VYTLPP**C**RD**K**LTKNQVSL**W**CLVKGFYPSDIAVEWESNGQPENNYKTTPPVLDSDGSFFLYSKL
TVDKSRWQQGNVFSCSVMHEALHNHYTQKSLSLSPGKGGGGGGGGGGGGGGGGGGGDKT
HTCPPCPAPELLGGPSVFLFPPKPKDTLMISRTPEVTCVVVDVSHEDPEVKFNWYVDGVEVHNA
KTKPREEQYNSTYRVVSVLTVLHQDWLNGKEYKCKVSNKALPAPIEKTISKAKGQPREPQVYT
LPPSRDELTKNQVSLTCLVKGFYPSDIAVEWESNGQPENNYKTTPPVL**K**SDGSFFLYS**D**LTVDK
SRWQQGNVFSCSVMHEALHNHYTQKSLSLSPG*

Short chain (knob and holes sequences in blue, electrostatic steering mutations in purple)

DKTHTCPPCPAPELLGGPSVFLFPPKPKDTLMISRTPEVTCVVVDVSHEDPEVKFNWYVDGVEV
HNAKTKPREEQYNSTYRVVSVLTVLHQDWLNGKEYKCKVSNKALPAPIEKTISKAKGQPREPQ
V**C**TLPPSRDELTKNQVSL**S**C**A**V**D**GFYPSDIAVEWESNGQPENNYKTTPPVLDSDGSFFL**V**SKLT
VDKSRWQQGNVFSCSVMHEALHNHYTQKSLSLSPG*

Fc3Y (AA/P) (The C1q “AA” mutations are indicated in red, the FcγRIIB mutation “P” in green)

Long chain

DKTHTCPPCPAPELLGGPSVFLFPPKPKDTLMISRTPEVTCVVVDVSHEDPEVKFNWYVDGVEV
HNAKTKP**P**EEQYNSTYRVVSVLTVLHQDWLNGKEYKCKVSN**A**ALPAPI**A**KTISKAKGQPREPQ
VYTLPPCRDKLTKNQVSLWCLVKGFYPSDIAVEWESNGQPENNYKTTPPVLDSDGSFFLYSKLT
VDKSRWQQGNVFSCSVMHEALHNHYTQKSLSLSPGKGGGGGGGGGGGGGGGGGGGDKTH
TCPPCPAPELLGGPSVFLFPPKPKDTLMISRTPEVTCVVVDVSHEDPEVKFNWYVDGVEVHNAK
TKP**P**EEQYNSTYRVVSVLTVLHQDWLNGKEYKCKVSN**A**ALPAPI**A**KTISKAKGQPREPQVYTL
PPSRDELTKNQVSLTCLVKGFYPSDIAVEWESNGQPENNYKTTPPVLKSDGSFFLYSDLTVDKS
RWQQGNVFSCSVMHEALHNHYTQKSLSLSPG*

Short chain (The C1q "AA" mutations are indicated in red, the FcγRIIB mutation "P" in green)

DKTHTCPPCPAPELLGGPSVFLFPPKPKDTLMISRTPEVTCVVVDVSHEDPEVKFNWYVDGVEV
HNAKTKP**P**EEQYNSTYRVVSVLTVLHQDWLNGKEYKCKVSN**A**ALPAPI**A**KTISKAKGQPREPQ
VCTLPPSRDELTKNQVSLSCAVDGFYPSDIAVEWESNGQPENNYKTTPPVLDSDGSFFLVSKLT
VDKSRWQQGNVFSCSVMHEALHNHYTQKSLSLSPG*

Fc3Y (FT/P) (The C1q "FT" mutations are indicated in red, the FcγRIIB mutation "P" in green)

Long chain

DKTHTCPPCPAPELLGGPSVFLFPPKPKDTLMISRTPEVTCVVVDVS**F**EDPEVKFNWYVDGVEV
HNAKTKP**P**EEQYNSTYRVVSVLTVLHQDWLNGKEYKCKV**T**NKALPAPIEKTISKAKGQPREPQ
VYTLPPCRDKLTKNQVSLWCLVKGFYPSDIAVEWESNGQPENNYKTTPPVLDSDGSFFLYSKLT
VDKSRWQQGNVFSCSVMHEALHNHYTQKSLSLSPGKGGGGGGGGGGGGGGGGGGDKTH
TCPPCPAPELLGGPSVFLFPPKPKDTLMISRTPEVTCVVVDVS**F**EDPEVKFNWYVDGVEVHNAK
TKP**P**EEQYNSTYRVVSVLTVLHQDWLNGKEYKCKV**T**NKALPAPIEKTISKAKGQPREPQVYTL
PPSRDELTKNQVSLTCLVKGFYPSDIAVEWESNGQPENNYKTTPPVLKSDGSFFLYSDLTVDKS
RWQQGNVFSCSVMHEALHNHYTQKSLSLSPG*

Short chain (The C1q "FT" mutations are indicated in red, the FcγRIIB mutation "P" in green)

DKTHTCPPCPAPELLGGPSVFLFPPKPKDTLMISRTPEVTCVVVDVS**F**EDPEVKFNWYVDGVEV
HNAKTKP**P**EEQYNSTYRVVSVLTVLHQDWLNGKEYKCKV**T**NKALPAPIEKTISKAKGQPREPQ
VCTLPPSRDELTKNQVSLSCAVDGFYPSDIAVEWESNGQPENNYKTTPPVLDSDGSFFLVSKLT
VDKSRWQQGNVFSCSVMHEALHNHYTQKSLSLSPG*

Fc3Y (EFT/P) (The C1q "EFT" mutations are indicated in red, the FcγRIIB mutation "P" in green)

Long chain

DKTHTCPPCPAPELLGGPSVFLFPPKPKDTLMISRTPEVTCVVVDV**EF**EDPEVKFNWYVDGVEV
HNAKTKP**P**EEQYNSTYRVVSVLTVLHQDWLNGKEYKCKV**T**NKALPAPIEKTISKAKGQPREPQ
VYTLPPCRDKLTKNQVSLWCLVKGFYPSDIAVEWESNGQPENNYKTTPPVLDSDGSFFLYSKLT
VDKSRWQQGNVFSCSVMHEALHNHYTQKSLSLSPGKGGGGGGGGGGGGGGGGGGDKTH
TCPPCPAPELLGGPSVFLFPPKPKDTLMISRTPEVTCVVVDV**EF**EDPEVKFNWYVDGVEVHNAK

TKP**P**EEQYNSTYRVVSVLTVLHQDWLNGKEYKCKV**T**NKALPAPIEKTISKAKGQPREPQVYTL
PPSRDELTKNQVSLTCLVKGFYPSDIAVEWESNGQPENNYKTTPPVLKSDGSFFLYSDLTVDKS
RWQQGNVFSCSVMHEALHNHYTQKSLSLSPG*

Short chain (The C1q “EFT” mutations are indicated in red, the FcγRIIB mutation “P” in green)
DKTHTCPPCPAPELLGGPSVFLFPPKPKDTLMISRTPEVTCVVVDV**EF**EDPEVKFNWYVDGVEV
HNAKTKP**P**EEQYNSTYRVVSVLTVLHQDWLNGKEYKCKV**T**NKALPAPIEKTISKAKGQPREPQ
VCTLPPSRDELTKNQVSLSCAVDGFYPSDIAVEWESNGQPENNYKTTPPVLDSDGSFFLVSKLT
VDKSRWQQGNVFSCSVMHEALHNHYTQKSLSLSPG*

Fc2 (-/-)
Long chain
DKTHTCPPCPAPELLGGPSVFLFPPKPKDTLMISRTPEVTCVVVDVSHEDPEVKFNWYVDGVEV
HNAKTKPREEQYNSTYRVVSVLTVLHQDWLNGKEYKCKVSNKALPAPIEKTISKAKGQPREPQ
VYTLPPCRDKLTKNQVSLWCLVKGFYPSDIAVEWESNGQPENNYKTTPPVLDSDGSFFLYSKLT
VDKSRWQQGNVFSCSVMHEALHNHYTQKSLSLSPGKGGGGGGGGGGGGGGGGGGGDKTH
TCPPCPAPELLGGPSVFLFPPKPKDTLMISRTPEVTCVVVDVSHEDPEVKFNWYVDGVEVHNAK
TKPREEQYNSTYRVVSVLTVLHQDWLNGKEYKCKVSNAALPAPIAKTISKAKGQPREPQVYTL
PPCRDKLTKNQVSLWCLVKGFYPSDIAVEWESNGQPENNYKTTPPVLDSDGSFFLYSKLTVDK
SRWQQGNVFSCSVMHEALHNHYTQKSLSLSPG

Short chain
DKTHTCPPCPAPELLGGPSVFLFPPKPKDTLMISRTPEVTCVVVDVSHEDPEVKFNWYVDGVEV
HNAKTKPREEQYNSTYRVVSVLTVLHQDWLNGKEYKCKVSNKALPAPIEKTISKAKGQPREPQ
VCTLPPSRDELTKNQVSLSCAVDGFYPSDIAVEWESNGQPENNYKTTPPVLDSDGSFFLVSKLT
VDKSRWQQGNVFSCSVMHEALHNHYTQKSLSLSPG

Fc2 (AA/-)
Long chain (The C1q “AA” mutations are indicated in red)
DKTHTCPPCPAPELLGGPSVFLFPPKPKDTLMISRTPEVTCVVVDVSHEDPEVKFNWYVDGVEV
HNAKTKPREEQYNSTYRVVSVLTVLHQDWLNGKEYKCKVSN**A**ALPAPI**A**KTISKAKGQPREPQ

VYTLPPCRDKLTKNQVSLWCLVKGFYPSDIAVEWESNGQPENNYKTTPPVLDSDGSFFLYSKLT
VDKSRWQQGNVFSCSVMHEALHNHYTQKSLSLSPGKGGGGGGGGGGGGGGGGGGGDKTH
TCPPCPAPELLGGPSVFLFPPKPKDTLMISRTPEVTCVVVDVSHEDPEVKFNWYVDGVEVHNAK
TKPREEQYNSTYRVVSVLTVLHQDWLNGKEYKCKVSN**A**ALPAPI**A**KTISKAKGQPREPQVYTL
PPCRDKLTKNQVSLWCLVKGFYPSDIAVEWESNGQPENNYKTTPPVLDSDGSFFLYSKLTVDK
SRWQQGNVFSCSVMHEALHNHYTQKSLSLSPG

Short chain (The C1q “AA” mutations are indicated in red)
DKTHTCPPCPAPELLGGPSVFLFPPKPKDTLMISRTPEVTCVVVDVSHEDPEVKFNWYVDGVEV
HNAKTKPREEQYNSTYRVVSVLTVLHQDWLNGKEYKCKVSN**A**ALPAPI**A**KTISKAKGQPREPQ
VCTLPPSRDELTKNQVSLSCAVDGFYPSDIAVEWESNGQPENNYKTTPPVLDSDGSFFLVSKLT
VDKSRWQQGNVFSCSVMHEALHNHYTQKSLSLSPG

Fc2 (FT/-)
Long chain (The C1q “FT” mutatios are indicated in red)
DKTHTCPPCPAPELLGGPSVFLFPPKPKDTLMISRTPEVTCVVVDVS**F**EDPEVKFNWYVDGVEV
HNAKTKPREEQYNSTYRVVSVLTVLHQDWLNGKEYKCKV**T**NKALPAPIEKTISKAKGQPREPQ
VYTLPPCRDKLTKNQVSLWCLVKGFYPSDIAVEWESNGQPENNYKTTPPVLDSDGSFFLYSKLT
VDKSRWQQGNVFSCSVMHEALHNHYTQKSLSLSPGKGGGGGGGGGGGGGGGGGGGDKTH
TCPPCPAPELLGGPSVFLFPPKPKDTLMISRTPEVTCVVVDVSFEDPEVKFNWYVDGVEVHNAK
TKPREEQYNSTYRVVSVLTVLHQDWLNGKEYKCKVTNKALPAPIEKTISKAKGQPREPQVYTL
PPCRDKLTKNQVSLWCLVKGFYPSDIAVEWESNGQPENNYKTTPPVLDSDGSFFLYSKLTVDK
SRWQQGNVFSCSVMHEALHNHYTQKSLSLSPG

Short chain (the C1q “FT” mutations are indicated in red)
DKTHTCPPCPAPELLGGPSVFLFPPKPKDTLMISRTPEVTCVVVDVS**F**EDPEVKFNWYVDGVEV
HNAKTKPREEQYNSTYRVVSVLTVLHQDWLNGKEYKCKV**T**NKALPAPIEKTISKAKGQPREPQ
VCTLPPSRDELTKNQVSLSCAVDGFYPSDIAVEWESNGQPENNYKTTPPVLDSDGSFFLVSKLT
VDKSRWQQGNVFSCSVMHEALHNHYTQKSLSLSPG

Fc2 (EFT/-)

Long chain (The C1q "EFT" mutations are indicated in red)

DKTHTCPPCPAPELLGGPSVFLFPPKPKDTLMISRTPEVTCVVVDV**EF**EDPEVKFNWYVDGVEV
HNAKTKPREEQYNSTYRVVSVLTVLHQDWLNGKEYKCKV**T**NKALPAPIEKTISKAKGQPREPQ
VYTLPPCRDKLTKNQVSLWCLVKGFYPSDIAVEWESNGQPENNYKTTPPVLDSDGSFFLYSKLT
VDKSRWQQGNVFSCSVMHEALHNHYTQKSLSLSPGKGGGGGGGGGGGGGGGGGGGDKTH
TCPPCPAPELLGGPSVFLFPPKPKDTLMISRTPEVTCVVVDV**EF**EDPEVKFNWYVDGVEVHNAK
TKPREEQYNSTYRVVSVLTVLHQDWLNGKEYKCKV**T**NKALPAPIEKTISKAKGQPREPQVYTL
PPCRDKLTKNQVSLWCLVKGFYPSDIAVEWESNGQPENNYKTTPPVLDSDGSFFLYSKLTVDK
SRWQQGNVFSCSVMHEALHNHYTQKSLSLSPG

Short chain (The C1q "EFT" mutations are indicated in red)

DKTHTCPPCPAPELLGGPSVFLFPPKPKDTLMISRTPEVTCVVVDV**EF**EDPEVKFNWYVDGVEV
HNAKTKPREEQYNSTYRVVSVLTVLHQDWLNGKEYKCKV**T**NKALPAPIEKTISKAKGQPREPQ
VCTLPPSRDELTKNQVSLSCAVDGFYPSDIAVEWESNGQPENNYKTTPPVLDSDGSFFLVSKLT
VDKSRWQQGNVFSCSVMHEALHNHYTQKSLSLSPG

Fc1 (-/-)

EPKSCDKTHTCPPCPAPELLGGPSVFLFPPKPKDTLMISRTPEVTCVVVDVSHEDPEVKFNWYV
DGVEVHNAKTKPREEQYNSTYRVVSVLTVLHQDWLNGKEYKCKVSNKALPAPIEKTISKAKG
QPREPQVYTLPPSRDELTKNQVSLTCLVKGFYPSDIAVEWESNGQPENNYKTTPPVLDSDGSFF
LYSKLTVDKSRWQQGNVFSCSVMHEALHNHYTQKSLSLSPGK**

Fc1 (AA/-) (The C1q "AA" mutations are indicated in red)

EPKSCDKTHTCPPCPAPELLGGPSVFLFPPKPKDTLMISRTPEVTCVVVDVSHEDPEVKFNWYV
DGVEVHNAKTKPREEQYNSTYRVVSVLTVLHQDWLNGKEYKCKVSN**A**ALPAPI**A**KTISKAKG
QPREPQVYTLPPSRDELTKNQVSLTCLVKGFYPSDIAVEWESNGQPENNYKTTPPVLDSDGSFF
LYSKLTVDKSRWQQGNVFSCSVMHEALHNHYTQKSLSLSPGK**

Supplemental Data Material and Methods

Complement dependent cytotoxicity (CDC) Assay with AQP4 expressing cells

The assay was slightly modified from Tradtrantip et al. [1]. The CDC assay was conducted using AQP4-expressing CHO cell line. AQP4-expressing CHO cells were labelled by incubation with 1mM Calcein-AM viability dye (eBioscience) for one hour, and then washed. Afterwards cells were incubated for 30 min with 1 µg/ml anti-AQP4-IgG1 and washed extensively. Fc3Y (-/-), Fc3Y (-/P) and CSL777 diluted in GVB2+ buffer mixed with 20% NHS (as complement source) were added to AQP4-IgG-coated cells for 15 minutes at RT and reaction stopped by adding 20 mM EDTA. Cells were centrifuged and supernatant collected and free Calcein measured.

C3b deposition human umbilical cord endothelial cells (HUVEC)

The assay was conducted according to Spirig et al. [2]. HUVECs were cultured according to the manufacturer's description (Lonza, Visp, Switzerland). For analysis of C3b deposition, HUVECs were opsonized with an anti-CD105 (Endoglin) mAb (MEM-226; ab60902; Abcam; RRID:AB_940842**)** before incubation with 20% NHS diluted in GVB2+ (1:5) for 30–60 min at 37°C. C3b deposition was detected using an FITC-conjugated anti-C3c polyclonal Ab (F0201; Dako; RRID:AB_2335709) and quantified by FACS.

References

1. Tradtrantip, L.; Felix, C.M.; Spirig, R.; Morelli, A.B.; Verkman, A.S. Recombinant IgG1 Fc Hexamers Block Cytotoxicity and Pathological Changes in Experimental in Vitro and Rat Models of Neuromyelitis Optica. *Neuropharmacology* **2018**, *133*, 345–353, doi:10.1016/j.neuropharm.2018.02.002.

2. Spirig, R.; Campbell, I.K.; Koernig, S.; Chen, C.-G.; Lewis, B.J.B.; Butcher, R.; Muir, I.; Taylor, S.; Chia, J.; Leong, D.; et al. rIgG1 Fc Hexamer Inhibits Antibody-Mediated Autoimmune Disease via Effects on Complement and FcγRs. *J. Immunol.* **2018**, *200*, 2542–2553, doi:10.4049/jimmunol.1701171.
